# Homœopathy

**Published:** 1855-09

**Authors:** 


### EDITORIAL.

*Homœopathy.*—In the August number, we inserted an article from a correspondent on this trite subject. We gave it a place, *first*, because it very fairly represented the leading doctrines of that *pathy*, as they were originally promulgated by Hahnemann and his immediate followers. Second, because it will enable those of our readers who are familiar with homœopathic practice at the present day, to judge how far it deviates from the original theories and practices of that sect. And third, because it would furnish an occasion for some remarks of our own, which may not *be* wholly unprofitable to the reader.

As our correspondent states, Homœopathy, as promulgated by Hahnemann and his followers, was based on two fundamental and distinct propositions, both of which were claimed as essential to the system. The first was, that "like cures like," or *similia similiabus curantur*; and the second, that the curative powers of medicines increase in a direct ratio with their dilution or attenuation, and consequently that none but *infinitesimal* doses are proper in the treatment of disease. The first of these propositions was not original with Hahnemann, nor with the sect called Homœopathists. The second was, however, and it was his bold and pertinacious promulgation of that idea, coupled with an unsparing denunciation of all who did not assent to its correctness, which led to his separation from the regular profession. So essential was this doctrine of *infinitesimal doses* considered, that nearly all the controversy that resulted from the first introduction of the system into practice was had in relation to it, while the first named proposition, *similia similiabus curantur*, attracted comparatively little attention. And our correspondent states truly when he asserts, that by the early and eminent of the Homœopathic sect, the *thirtieth* dilution or attenuation was considered the lowest that ought to be used in practice. But a very few extracts from the writings of living members of the sect, will show how far they have fallen from the high *spiritual* position of their original leaders. Thus Dr. B. F. Bowers of New York, in an article published in the *Homœopathic Examiner*, says: "He who takes as his guide in practice, the law, *like cures like*, whether he *gives much* or *little medicine*, *large* or *small doses*, is in principle a homœopathist, while he who rejects this law rejects homœopathy. Those persons who confound homœopathy with infinitesimal doses overlook an obvious distinction."—Again the same writer says: "Every homœopathist will agree, that the dose *must be large enough* to produce the required effect; more than this is at least unnecessary; and may be injurious. What is a suitable dose must be left to the enlightened judgement and experience of the practitioner." This amounts to a full surrender of one of the most prominent and cardinal doctrines of the original homœopathic school The language is: "*The dose must be large enough to produce the required effect;*" thus coming directly back to the common sense of the regular profession. Nor is it in theory alone that the greater number of the homœopathists in this country have abandoned the infinitesimal doses In looking over their journals, wherever prescriptions are given in detail, they very rarely go beyond the sixth dilution, and in a large proportion of the cases it ranges from the first to the third. Thus, in a late number of the *Chicago Homœopath*, for a slight case of Chorea, we find the following: "Prescribed Cuprum Aceticum, 3d, *two grains* every morning, an hour after breakfast, with fresh air and exercise" And again, for a case of simple hoarseness or partial aphonia, "Prescribed Phosphorus, 6th, four pills three times a day." Here is no putting a pellet of the 30th attenuation into a tumbler of distilled water, and allowing the patient to take a teaspoonful once in 24 or 48 hours; nor the putting of a drop of the 60th dilution into an ounce of water, and directing the patient to *smell* it once or twice a day, as was practiced by Hahnemann and his immediate followers.

The inference from all this, and much more that might be adduced, is very plain and pregnant with meaning. It is, that the proposition to increase the *power* of medicine by diminishing the dose to *infinitesimality* is virtually abandoned both in theory and practice. And any homœopathist may give "much or little medicine, large or small doses," as his "judgment and experience may dictate." Then there is nothing left of homœopathy but the pretended law, "*like cures like.*" And as the selection of remedies in accordance with this proposition depends entirely on the judgment or fancy of each individual practitioner, it is obvious that, while adhering to the name of Homœopathy and making a show of sugar pellets, they are practically owing what success they do have to the use of ordinary remedies in not *very* attenuated doses. For, if the principle, "like cures like" will sanction the prescription of phosphorus for aphonia, and acetate of copper for chorea, there is no active principle in the materia medica that may not be prescribed for any given disease. There is one error that homœopathists most industriously propagate in the public mind, viz., that the only reason why their system, as they style it, is not universally adopted is, because the old, "hunkerish propensities and prejudices" of the regular profession will not allow the members to investigate its merits. Now every decently intelligent homœopathist in the country knows full well that their system was most carefully investigated, both theoretically and practically, by members of the most learned medical societies in the world. That committees were appointed on it, particular patients in hospitals assigned for its practical application, and patient, detailed reports made; all of which only proved the system, as promulgated by Hahnemann, with its *infinitesimal* principle attached, entirely inefficient in practice and unworthy of further attention. And besides these public investigations, it is probable that for ten years after the first promulgation of the speculations of Hahnemann, very few intelligent physicians failed to put his fancies to the test of practical application, more or less. I remember full well that I tried it on more than one case of chronic disease, and patiently for many weeks tested the virtues of medicines on myself, strictly according to the directions of the great lights of the system. Refuse to investigate! why, does anybody suppose that physicians are such fools that they would pay out $100 per annum, perhaps, for quinine to use in their practice, if by any process of division, dilution or attenuation of doses, they could make *one dollar's* worth last all the year? But let our homœopathic friends continue to progress in the same direction a few years longer, and they will scarcely acknowledge more obedience to the absurd proposition, "like cures like," than they now do to the original *infinitesimal* humbug of their great founder.

